# Iodine nutritional status of women in their first trimester of pregnancy in Catalonia

**DOI:** 10.1186/s12884-017-1423-4

**Published:** 2017-07-26

**Authors:** Maria Teresa Torres, Lidia Francés, Lluis Vila, Josep María Manresa, Gemma Falguera, Gemma Prieto, Roser Casamitjana, Pere Toran, the IODEGEST study group, Montse Abella, Nuria Sampedro, Glòria Miralpeix, Montse Villanueva, Concepción Manzano, Judit Cos, Pilar Soteras, Fermina Casas, Coloma Graells, Mireia Llucià, Rosalia Ibars, Encarna López, Montserrat Manzanares, Irene Lorente, Eva Artieda, Meritxell Casajoana, Dolors Muñoz, Llucia Burgos, Angelica Hidalgo, Anna Fusté, Dolors Lladó, Rosa Subirats, Angelina Masoliver, Imma Trujillo, Rosa Banús, Dolors Salas, Montse Pujol, Dolors Grau, Roser Sanglas, Anabel Mayos, Náyade Crespo, Rosa Codina, Rosa Forn, Montserrat Galí, Antonia Hidalgo, Teresa Macià, Mercè Vendrell, Montserrat Sallent, Montserrat Ribera, Rosa Oller, Teresa Riba, Esther Romero, Adelaida Expósito, Encarnació Santaeulàlia, Anna Vilaseca, Dolors Guix, Gemma Olivera, Merche García, Rosa Sans, Marta Roman, Mª Jose Vila, Maite Martinez, Esther Serrano, Sonia Díaz, Carolina Alcaine, Susana Sancho, Remei Fenollosa, Encarna Gascón, Núria Risques, Araceli Santamaria, Remei Corominas, Xavi Espada, Maria Helena Perez, Concepción de la Fuente, Assumpta Prats, Maria Rosa Cabedo, Carme Magem, Mercedes Vigil, Carmen Biern, Montse Bach, Joana Relat, Engracia Coll, Carmen Bayascas, Olga Ezquerro, Patricia Reategui, Anna Campos, Rosa Bach, Inés Molina, Eva Martinez, Anna Bartolí, Rosa Ferrer, Rocio Hernandez

**Affiliations:** 1 Atenció a la Salut Sexual i Reproductiva (ASSIR), CAP Antoni Creus i Querol, Institut Català de la Salut, Terrassa, Barcelona Spain; 2 Unitat de Suport a la Recerca Metropolitana Nord, Institut Universitari d’Investigació en Atenció Primària Jordi Gol (IDIAP Jordi Gol), Sabadell, Barcelona Spain; 3 GRASSIR research group, IDIAP Jordi Gol, Generalitat de Catalunya, Barcelona, Spain; 4 Departament d’Infermeria, Universitat Autònoma de Barcelona, Bellaterra, Cerdanyola del Vallès Spain; 5 Departament d’Infermeria, Universitat de Barcelona, L’Hospitalet de Llobregat, Barcelona Spain; 6 Servicio de Endocrinología y Nutrición, Hospital de Sant Joan Despí ‘Moisès Broggi’, Sant Joan Despí, Barcelona Spain; 7 Atenció a la Salut Sexual i Reproductiva (ASSIR) Gerència Territorial Metropolitana Nord, Institut Català de la Salut, Sabadell, Barcelona Spain; 8 Gerencia de Atención Primaria, Ávila, Spain; 9 Centro de Diagnóstico Biomédico – Bioquímica y Genética Molecular, Hospital Clínic, Barcelona, Spain

## Abstract

**Background:**

Sufficient iodine intake is needed during pregnancy to ensure proper fetal development. The iodine levels of women in their first trimester of pregnancy in Catalonia are currently unknown. This data would help to determine whether our public health services should establish recommendations or interventions in this line. The aim of this study was to investigate the iodine nutritional status, prevalence of urinary iodine <150 μg/L, and tobacco use in the first trimester of pregnancy in our setting.

**Methods:**

Cross-sectional study. Data were collected during 2008–2009 from women in their first trimester at the primary care centers of the province of Barcelona (Spain). Pregnant women included in the study completed a questionnaire on eating habits and underwent urinary iodine concentration (UIC) assessment.

**Results:**

Nine hundred forty five women completed the dietary questionnaire and urinary iodine testing. Median UIC was 172 μg/L, with 407 participants (43.1%) showing levels <150 μg/L. On multivariate logistic regression analysis, intake of 1–2 glasses of milk per day, OR = 0.636 95% CI (0.45–0.90) or >2 glasses, OR = 0.593 95% CI (0.37–0.95); iodized salt consumption, OR = 0.678 95% CI (0.51–0. 90); and use of iodine supplementation, OR = 0.410 95% CI (0.31–0.54), protected against the risk of UIC <150 μg/L. Simultaneous consumption of iodized salt and milk (≥1 glass/day) showed a larger protective effect: OR = 0.427, 95% CI (0.31–0.54).

**Conclusion:**

The median UIC of the pregnant women surveyed indicated an acceptable iodine nutritional status according to the criteria established by the WHO and ICCIDD. The risk of urinary iodine <150 μg/L decreased with simultaneous consumption of milk and iodized salt, similar to the decrease seen with iodine supplementation.

**Electronic supplementary material:**

The online version of this article (doi:10.1186/s12884-017-1423-4) contains supplementary material, which is available to authorized users.

## Background

A recent nationwide study conducted in Spain has reported that iodine nutrition is adequate in the general population, which showed a median urinary iodine concentration (UIC) of 117 μg/L [1]. Nonetheless, in the subgroup of women of childbearing age, median UIC was 114 μg/L, a value indicating a situation of risk in pregnancy [1]*.*

Iodine is an essential micronutrient that must be supplied by regular intake in the food consumed. It has a vital function in the synthesis of thyroid hormones, which act on several organs and body systems, and is especially important for central nervous system development starting from the earliest stages of embryonic and fetal growth [2].

Iodine requirements vary with age and the individual’s physiological condition. In pregnancy, thyroid metabolism undergoes a series of changes as the demand for iodine increases due to embryonic and fetal development. Since the early stages of pregnancy, the thyroid gland undergoes stimulation due to the effect of chorionic gonadotropin hormone [3]. There is an increase in the available blood volume and the iodine distribution space, iodine is transferred from the mother’s circulatory system to the fetal-placental unit, and an increase in glomerular filtration leads to increasingly larger amounts of iodine being eliminated in urine (30%–50%). This combination of factors results in a high demand for iodine during pregnancy [4–6]. During the first trimester, thyroid hormones for embryonic and fetal tissues rely exclusively on the available maternal hormones; hence, iodine deficiency in the mother may have a negative impact on prenatal development [7]. Clinical studies have reported a relationship of iodine deficiency and maternal hypothyroxinemia during pregnancy with impaired neurocognitive and psychological development of the children [3, 8–13]. Therefore, it is important to increase iodine intake starting in the early stages of pregnancy and, if possible, even beforehand. The World Health Organization (WHO), together with the United Nations International Children’s Emergency Fund (UNICEF) and the International Council for Control of Iodine Deficiency Disorders (ICCIDD), recommend a daily iodine intake of between 200 and 250 μg/day during pregnancy [14].

Under normal conditions, there is a balance between iodine intake and urinary output, making the UIC a good indicator of recently consumed iodine [15]. The median UIC indicating optimum iodine intake in pregnant women is 150 to 250 μg/L [14, 15]. Studies carried out in various European countries, including Spain, have reported highly variable values, ranging from 60 to 250 μg/L [16].

Several strategies can be applied to ensure an adequate iodine supply and to correct deficiencies in this regard. The WHO and other international agencies have advocated universal salt iodization for worldwide consumption. In cases where iodine requirements cannot be guaranteed through diet during pregnancy, the WHO recommends potassium iodide supplementation [14].

Another aspect to include when considering the dietary and health habits of pregnant women is tobacco use and its impact on maternal thyroid function. Tobacco is considered a goitrogenic substance, as it inhibits thyroid uptake of iodine [17]. Tobacco use during pregnancy has been associated with changes in thyroid function in both the mother and fetus [18–20], and with changes in the UIC [21]. During lactation, tobacco use lowers the iodine content in breast milk [22].

Currently, there is little available data on the iodine nutritional status in pregnant woman in Catalonia (an autonomous region in northeast Spain) or the dietary habits that may be determinant in this regard. The available information comes from studies conducted in limited areas of our region or in women in the third term of pregnancy [23, 24]. To fill this gap, the aim of this study was to determine the iodine status of a population of pregnant women during the first trimester of pregnancy—the time when thyroid deficiency can have the greatest impact on embryonic development—and to investigate the habits affecting the iodine status in this population in a large geographical area of Catalonia. The ultimate aim was to gain information that will be useful to assess the need for interventions in this population by a public health program.

## Methods

### Design

This is a cross-sectional descriptive study, based on information collected from women in their first trimester of pregnancy in 2008–2009. The information was obtained within the structure of a clinical trial whose ultimate purpose is to evaluate the effect of an intervention on eating habits and its benefits on iodine levels in pregnant women [25]. The present report describes the iodine status results in this population. The study was approved by the Clinical Research Ethics Committee at the *Primary Care Research Institute* (IDIAP) Jordi Gol.

### Setting

The study was conducted within the framework of the primary care center Program for Sexual and Reproductive Health Care (PASSIR) of the *Catalonia Central* and *Metropolitana Nord* Regional Offices of the *Institut Català de la Salut* (ICS, Catalonian Institute of Health) in the province of Barcelona (Spain).

### Participants

A consecutive recruitment was carried out within a clinical trial framework. During 2008 and 2009, all women older than 17 years who were seen in the participating centers in their first trimester of pregnancy (<13 weeks) and accepted to participate were included in the study. Pregnant women with thyroid disease, no telephone contact, or difficulty communicating with the health personnel (cognitive, sensory, or language problems), and those refusing to participate, were excluded. A calculated sample size of 989 pregnant women was needed to answer the hypothesis of the clinical trial [25].

### Data collection

The socio-demographic data included patient age, place of origin, place of residence (rural/urban), and educational level. Information on dietary and other habits was collected at a personal interview by midwives in the participating primary care centers, using a standardized questionnaire [25] showing good reliability (Cronbach’s alpha, 0.960 and intraclass correlation coefficient, 0.927). The questionnaire contained items related to consumption of cow milk (glasses/day; 1 glass = 200 mL), and fish (servings/week), regular consumption of iodized salt (Yes/No), daily use of iodine supplements (potassium iodide or iodine vitamin supplements) (Yes/No), use of iodinated antiseptics, and tobacco use.

Urinary iodine concentration (μg/L) was determined as follows: A first morning urine sample was collected from each woman, quickly frozen at −40 °C, and transported within 24 to 48 h to a central laboratory (Barcelona Hospital Clinic), where UIC determination was performed using the Benotti & Benotti method [26]. Urine was first digested with chloric acid and then underwent the Sandell-Kolthoff reaction, in which iodine was determined by its action as a catalyst in the reduction of ceric ammonium sulphate in the presence of arsenious acid. The inter-assay and intra-assay coefficients of variation of the technique were 15.5% and 12.6%, respectively. Three times a year, the UIC assay undergoes evaluation by an external quality assessment program from the Spanish Association of Neonatal Screening (AECNE). UIC values were dichotomized as <150 μg/L (insufficient) and ≥150 μg/L (adequate) [14] in the analyses.

### Statistical analysis

Quantitative variables are described as the mean and standard deviation or the median and first and third quartiles (Q1-Q3) for those with a non-normal distribution. Categorical variables are expressed as the absolute frequency and percentage.

The Student *t* test for independent data and the Mann-Whitney *U* test, as appropriate, were used for quantitative variables and the Pearson chi-square test for categorical variables.

A multivariate logistic regression model was performed, in which the dependent variable was UIC less than or equal to 150 μg/L. The Initial model contained all the covariates that individually had an association with UIC according to a significance level of *p* ≤ 0.1. The final model included statistically significant (*p* < 0.05) covariates, while also considering Akaike’s information criterion and biological plausibility. Akaike’s information criterion is a tool that balances the information provided by a set of variables (goodness of fit) and the principle of parsimony (use of the minimum possible set of variables, less complexity), making it useful for model selection.

All analyses were performed with SPSS for Windows, version 22.0. Significance was set at a *p*-value of ≤0.05.

## Results

In total, 985 pregnant women in their first trimester of pregnancy were recruited. We were able to obtain the urinary iodine concentration in 970 (98.5%) women, and 945 (95.6%) also answered the standardized questionnaire.

### Characteristics of the sample

The mean age of the participants was 30.6 (4.6) years, and 784 (83.0%) were Spanish. Most (696, 73.7%) were from urban areas. The breakdown by educational level was as follows: 5 (0.5%) had received no formal education, 28 (3.0%) had not completed primary school, 232 (24.6%) had completed primary school, 393 (41.6%) had completed secondary school, and 287 (30.4%) had a university degree. As to tobacco use, 221 (23.4%) said that they were smokers and 98 (10.4%) ex-smokers (had stopped smoking less than 1 year previously, including those who stopped in early pregnancy); 176 (20.4%) women continued smoking during the first trimester of pregnancy.

### Urinary iodine concentration

**Fig. 1:**
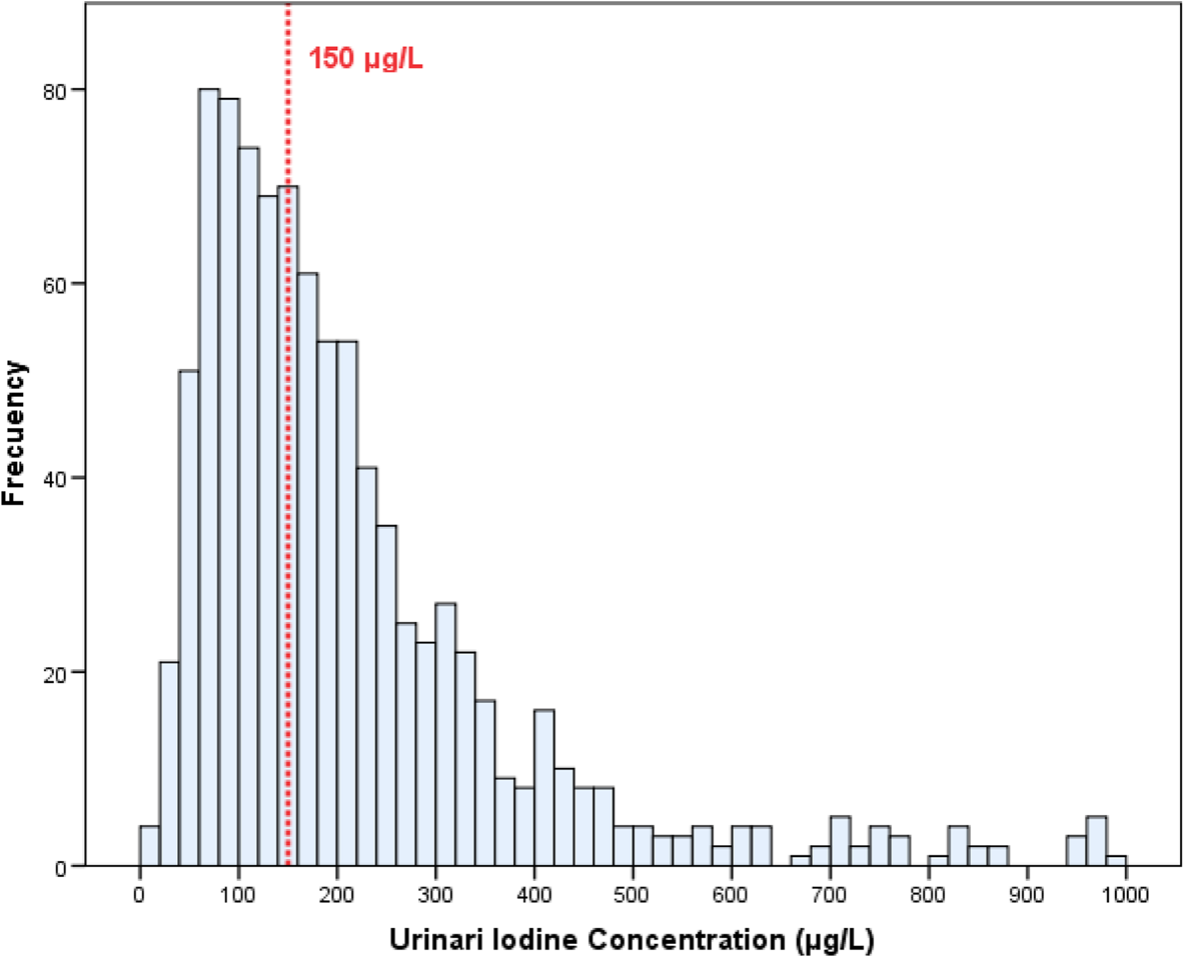
Histogram of the iodine concentration in the urine of pregnant women in their first trimester of pregnancy. The discontinuous line indicates the value of urinary iodine recommended in pregnant women (≥150 mg/dL). The 43.1% of these women had UIC <150 mg/dL

The median overall UIC [Q1-Q3] was 172 μg/L [103.8–289.3]. UIC distribution is depicted in Fig. 1. Among the total of participants, 43.1% had a UIC <150 μg/L and 8.8% a UIC >600 μg/L. Median UIC did not differ according to the women’s place of residence (rural/urban), place of origin, education level, use of iodinated antiseptics (3.1% of the population), or tobacco use (Table 1). The Spearman rho correlation coefficient between the number of cigarettes smoked and UIC level was nil (*r* = −0.074, *p* = 0.386). In total 337 (35.7%) women reported using iodized salt. The median UIC was higher in this group than in nonusers (189 vs 158 μg/L; *p* < 0.001) (Table 2). The 442 (46.8%) women taking iodine supplementation also showed a higher median UIC than those who did not (209.5 vs 140 μg/L; *p* < 0.001 We found that an increase in milk consumption was associated with a higher UIC (139.5, 176, and 198 μg/L for 0, 1–2, and >2 glasses/day, respectively; *p* = 0.003). The median UIC showed no differences in relation to fish consumption.

**Table 1:** Characteristics of pregnant women and related urinary iodine level

	Urinary Iodine ≥150 μg/L	Urinary Iodine <150 μg/L	*p* ^a^	Urinary iodine (μg/L) Median [Q3-Q3]	*p* ^b^
TRIMESTER 1	*n* = 556	*n* = 414		*n* = 970	
				172.0 [103.8–289.3]	---
Age	31.0 (4.5)	29.9 (4.6)	<0.001	---	---
Place of residence			0.526		0.976
Urban	413 (57.9%)	300 (42.1%)		157.5 [95.0–243.8]	
Rural	143 (556%)	114 (44.4%)		154.0 [100.6–231.5]	
Place of origin			0.127		0.335
Native of Spain	448 (57.1%)	336 (42.9%)		169.5 [102.2–287.5]	
South America	47 (66.2)	24 (33.8%)		192.5 [94.1–309.3]	
Africa	24 (46.2%)	28 (53.8%)		145.5 [95.3–211.3]	
Other	19 (50.0%)	19 (50.0%)		135.0 [85.0–249.0]	
Educational level			0.035		0.623
Illiterate	2 (40.0%)	3 (60.0%)		85.0 [47.6–204.0]	
Incomplete primary school	13 (46.4%)	15 (53.6%)		148.0 [79.8–333.5]	
Primary School	126 (54.3%)	106 (45.7%)		150.0 [103.5–238.5]	
High School	221 (56.2%)	172 (43.8%)		154.0 [95.5–232.0]	
University degree	176 (61.3%)	111 (38.7%)		163.0 [93.9–252.8]	
Tobacco use			0.253		0.263
Non-smoker	374 (59.1%)	259 (40.9%)		181.0 [104.0–294.0]	
Ex-smoker	54 (55.1%)	44 (44.9%)		168.0 [105.8–319.5]	
Smoker	123 (53.0%)	109 (47.0%)		157.5 [98.1–242.5]	

^a^Pearson’s chi-square test

^b^Mann-Whitney *U* test

**Table 2:** Iodine intake according to the questionnaire and related urinary iodine level

	Urinary Iodine ≥150 μg/L	Urinary Iodine <150 μg/L	*p* ^c^	Urinary iodine (μg/L) Median [Q1-Q3]	*p* ^d^
TRIMESTER 1	*N* = 538	*N* = 407		*N* = 945	
Milk^a^			0.016		0.003
0 glasses	85 (47.8%)	93 (52.2%)		139.5 [88.7–230.0]	
1–2 glasses	372 (58.8%)	261 (41.2%)		176.0 [105.0–294.3]	
> 2 glasses	81 (60.4%)	53 (39.6%)		198.0 [115.0–316.0]	
Fish^b^			0.220		0.926
Non-consumption	31 (53.4%)	27 (46.6%)		165.0 [95.8–336.0]	
1 serving	136 (55.5%)	109 (44.5%)		169.0 [104.0–282.5]	
2 servings	163 (56.2%)	127 (43.8%)		168.5 [98.9–307.0]	
3 servings	115 (57.5%)	85 (42.5%)		171.5 [104.2–261.2]	
> 3 servings	93 (61.2%)	59 (38.8%)		180.0 [101.2–295.0]	
Iodized salt			<0.001		0.001
No	321 (52.8%)	287 (47.2%)		158.0 [95.3–278.0]	
Yes	217 (64.4%)	120 (35.6%)		189.0 [116.5–305.0]	
Iodine supplements			<0.001		<0.001
No	234 (46.5%)	269 (53.5%)		140.0 [84.0–221.0]	
Yes	304 (68.8%)	138 (31.2%)		209.5 [131.0–342.0]	

^a^Glasses/day

^b^Servings/week

^c^Pearson’s chi-square test

^d^Mann-Whitney *U* test

### Association with UIC <150 μg/L

Urinary iodine values were dichotomized into <150 μg/L and ≥150 μg/L; 407 (43.1%) women had values below 150 μg/L (Tables 1 and 2). On bivariate analysis, UIC <150 μg/L showed no relationship with the women’s place of residence (urban or rural), place of origin, education level, or smoking habit. However, higher milk consumption (*p* = 0.016), iodized salt use (*p* < 0.001), and iodine supplementation (*p* < 0.001) were associated with UIC ≥150 μg/L (Table 2). No associations were found between the UIC value and fish consumption or exposure to iodinated antiseptics.

### Multivariate analysis

To determine which of the variables studied were associated with an insufficient UIC, multivariate logistic regression analysis was performed using UIC <150 μg/L as the dependent variable and each of the sociodemographic variables and items on the dietary questionnaire as independent variables.

In Model 1, milk consumption had a protective effect on UIC <150 mg/L, which increased with increasing milk consumption from 1 to 2 to >2 glasses/day (OR 0.636 [0.45–0.90] and 0.593 [0.37–0.95], respectively). Iodine supplementation and iodized salt intake also provided a protective effect (OR 0.410 [0.31–0.54] and OR 0.678 [0.51–0.90], respectively) (Table 3).

**Table 3:** Multivariate logistic regression analysis to determine the role of dietary intake and iodine supplementation on the risk of having an iodine level < 150 μg/L

	β	SE	OR	[95%CI]	*p*
MODEL 1					
Constant	0.633	---	---	---	---
Daily milk consumption					
No consumption	Reference	---	---		---
1–2 glasses	−0.453	0.176	0.636	[0.45–0.90]	0.010
> 2 glasses	−0.522	0.239	0.593	[0.37–0.95]	0.029
Iodized salt intake					
No consumption	Reference	---	---		---
consumption	−0.389	0.145	0.678	[0.51–0.90]	0.007
Iodine supplementation					
No consumption	Reference	---	---		---
consumption	−0.890	0.138	0.410	[0.31–0.54]	<0.001
MODEL 2					
Constant	0.617	---	---	---	---
Iodized salt intake and daily milk consumption					
No consumption	Reference	---	---		---
Only Iodized salt	−0.351	0.320	0.704	[0.38–1.32]	0.276
Only Milk	−0.445	0.216	0.641	[0.42–0.98]	0.039
Iodized salt and Milk	−0.850	0.236	0.427	[0.27–0.68]	<0.001
Iodine supplementation					
No consumption	Reference	---	---		---
consumption	−0.888	0.138	0.411	[0.31–0.54]	<0.001

Result of exploratory multivariate logistic regression analysis. The dependent variable was determined by the presence of UIC <150μg/L.

Model 1 shows the covariates that were statistically significant (*p* < 0.05), taking under consideration Akaike’s information criterion and biological plausibility

Model 2 was obtained by combining salt intake and milk in a single variable

In Model 2, daily consumption of 1 or more glasses of milk was combined with iodized salt use (Table 3). Consumption of iodized salt alone was a protective factor, but was non-significant, likely because of the relatively small number of women who used this product (OR 0.704 [0.38–1.32]; *p* = 0.276). However, intake of a single glass of milk yielded a protective effect (OR 0.641 [0.42–0.98]; *p* = 0.039), which was even greater if complemented with iodized salt consumption (OR 0.0.427 [0.27–0.66]; *p* < 0.001), in a manner similar to the effect of iodine supplementation (OR 0.411 [0.31–0.54]; *p* < 0.001).

## Discussion

The median UIC of pregnant women in their first trimester (172 μg/L) indicated an adequate iodine nutritional status (≥150 μg/L) [14], although 43.1% of the sample showed values <150 μg/L. This overall finding is consistent with the results obtained in studies conducted in adults and children (4 and 6 years old) from Catalonia, which reported UICs indicative of adequate iodine nutrition [27–29]. In our study, iodine status of the pregnant women included did not differ according to the place of residence (rural or urban), place of origin (native or immigrant), education level, or tobacco use.

The median UIC we found (172 μg/L) was notably higher than that obtained in a study performed in 2010 by Alvarez-Pedrerol et al. in Sabadell, an area within the setting of our study. In total, 600 women in the third trimester were included and the median UIC was 104 μg/L [23]. These differences may be explained by the lower consumption of iodized salt in that population compared to our cohort (11.5% vs 35.7%). In another study, including 267 pregnant women in the first trimester in two areas of Catalonia, 139 in a mountain area and 128 in coastal area, the median UIC was found to be higher (coastal area, 142 μg/L and mountain area 209 μg/L); iodized salt was used by 36.4% and 58% of the women, respectively [24].

In a study including a sample of 1844 women in the first trimester of pregnancy from 3 areas of Spain (Valencia, Guipúzcoa, Sabadell), the median UIC was lower than 150 μg/L in Valencia and Sabadell, but not in Guipúzcoa [30]. In a study conducted in Toledo (Spain) including 525 pregnant women, the median UIC was 164 μg/L [31]. The women were divided into 3 groups: 69 pregnant women who did not use iodized salt or iodine supplementation, 75 pregnant women who used iodized salt but not supplementation, and 381 pregnant women who used potassium iodide supplementation. Median UICs in the three groups were 134.5 μg/L, 146 μg/L, and 183 μg/L, respectively. In several studies, median UIC value >150 μg/L was associated with iodine supplementation [30–34].

In most European countries, very low median UICs have been reported, often less than 100 μg/L [14, 35], with the exception of Switzerland, Sweden, Slovenia, and some parts of Germany [36–40],

In the present study, UIC analysis in women who did not take potassium iodide supplements revealed that iodized salt intake along with milk consumption was sufficient to maintain median UIC above 150 μg/L (Table 3), although neither milk nor iodized salt intake alone showed a relationship with this value.

Multivariate analysis disclosed the independent roles of intake of milk, iodized salt, and supplementation on the UIC. An association was found between milk consumption and the UIC, and when iodized salt intake was combined with this variable, protection against iodine levels <150 μg/L was even higher, and similar to the protection obtained with supplementation (Table 3).

A similar association was detected in a recent study in northern Spain (Asturias): pregnant women who consumed 2 or more portions of dairy products per day had a UIC of 230 μg/L, whereas those who consumed less had a level of 191 μg/L. In addition, this study found a median UIC ≥150 mg/L in pregnant women who consumed iodized salt (41). In the study by Alvarez-Pedrerol et al., there was no association between the UIC and the foods included in their survey, with the exception of milk [23]. These authors also found that increases in milk consumption elicited a parallel increase in mean UIC values: 0 glasses/day, 78 μg/L; 1–2 glasses/day, 100 μg/L; and >2 glasses/day, 117 μg/L. A study in pregnant women conducted in the United Kingdom also revealed a significant positive association between milk consumption and the UIC [41]: The mean urinary iodine/urinary creatinine ratio value was 72 μg/g when milk consumption was <140 mL/day, which rose to 150 μg/g when consumption was more than 280 mL/day.

In Spain, the effect of milk consumption on the UIC was first detected in a study carried out in children (4–15 years old) more than 10 years ago [42]. Other studies including a population of adults and children who did not consume iodized salt, reported a median UIC of more than 100 μg/L [24, 43–45], suggesting that other sources of iodine in addition to iodized salt were being consumed, such as dairy products. A recent analysis of 362 samples of milk from different parts of Spain showed a mean iodine level of 259 (±58) μg/L [46]. Furthermore, in a sample of more than 4000 Spaniards aged 18 years and older, there was a significant association between milk consumption at least once a day and a UIC >100 μg/L [1]. Similar results were observed in a nationwide study conducted in a population of almost 2000 Spanish children [47]. The role of milk as a source of iodine, as was seen in Spain, is recognized in other European countries [48–53]. However, the “accidental iodination” of milk, such as that occurring in the United Kingdom [48], implies a risk of considerable variations in iodine content if there is no regulation and control. Australia has seen a drop in iodine nutrition due to a decreased iodine content in its milk supply [54, 55].

Iodized salt is the food traditionally recommended by the WHO and ICCIDD to ensure a proper level of iodine nutrition in the population [56]. The risk of iodine deficiency could be resolved if at least 90% of households consumed iodized salt. In Spain, however, iodized salt consumption in households is less than 50% and in the case of pregnant women, 35.5% [1]. As in other studies performed in our geographic area [23, 24], no association was found between fish consumption and the UIC in the pregnant women studied.

About 9% of our population had extreme UIC values (>600 μg/L). Although potassium iodide supplementation was higher in this group, it is difficult to attribute this to such a high UIC. However, seaweed consumption, application of iodized antiseptics in pregnancy [57], and use of salt with excessive iodination could justify UIC values >600 mg/dL.

Some studies have reported an association between smoking and an increased risk of goiter or other thyroid problems [18, 20, 24, 51, 58], but we did not find a significant effect of tobacco use on UIC values.

## Conclusion

In conclusion, the overall median UIC of women in their first trimester of pregnancy found in our study is indicative of adequate iodine nutritional status according to the criteria established by the WHO and ICCIDD, although 43.1% were found to have suboptimal levels. Simultaneous consumption of iodized salt and milk was as effective as potassium iodide supplementation to achieve adequate iodine nutrition. The iodine concentration in salt in Spain (60 ppm) and the high concentration of iodine in cow milk, which has increased in recent years, may explain these findings.

Women of childbearing age in our region should consume iodized salt and milk to maintain an optimal UIC, particularly those who are planning a pregnancy. A good intra-thyroidal deposit of iodine enables the gland to adapt and respond better to the physiologic changes that occur during pregnancy. If women use these iodine-rich products in the year before and during pregnancy, their iodine status will likely be adequate and iodine supplementation will not be necessary. Public health campaigns should be developed in our setting to promote consumption of iodized salt. Moreover, our national food agencies should ensure adequately iodized salt and regular monitoring of iodine content in milk.

## Additional file

Additional file 1. Iodegest Data Analysis. (XLSX 47 kb)
